# Associations between exposure to common technology devices and reported neck pain among Iranian school-age adolescents: a cross sectional study

**DOI:** 10.1186/s12891-023-07010-8

**Published:** 2023-11-13

**Authors:** Mohaddeseh Azadvari, Mojdeh Sarzaeim, Sarvin Rajabi, Alireza Yahyaee, Seyede Zahra Emami Razavi, Afarin Haghparast, Azam Biderafsh, Amin Nakhostin-Ansari, Maryam Hosseini, Masood Ghahvechi

**Affiliations:** 1 Department of Physical Medicine and Rehabilitation, Sina Hospital, Tehran University of Medical Sciences, Tehran, Iran; 2 Department of Physical Medicine and Rehabilitation, Imam Khomeini Hospital, Tehran University of Medical Sciences, Tehran, Iran; 3 School of Medicine, Tehran University of Medical Sciences, Poursina Avenue, Enqelab sq, Tehran, Iran; 4 Sports Medicine Research Center, Neuroscience Institute, Tehran University of Medical Sciences, Tehran, Iran; 5 Department of Epidemiology and BioStatistics, Health faculty, Tehran University of Medical Sciences, Tehran, Iran; 6 Pediatrics Center of Excellence, Department of Pediatric Neurology, Growth and Development Research Center, Children’s Medical Center, Tehran University of Medical Sciences, Tehran, Iran

**Keywords:** Adolescents, Neck pain, Prevalence, Technology devices

## Abstract

**Background:**

The advancement of technology has contributed to a more sedentary lifestyle, and the extensive use of handheld devices among adolescents may potentially result in neck pain. This study aimed to assess the association between exposure to common technology devices and self-reported neck pain in Iranian school-age adolescents.

**Methods:**

This cross-sectional study was conducted between June and October 2021, employing a randomized multi-stage cluster sampling approach. We enrolled 808 adolescent students aged 11 to 19 years old. We asked participants about any neck pain they experienced in the week leading up to the study. Additionally, we gathered demographic information and assessed participants’ use of electronic devices using a questionnaire.

**Results:**

Our study comprised 73.5% female participants with an average age of 15.1 ± 1.7 years and 26.5% male participants with an average age of 14.5 ± 1.5 years. In the regression model, the female gender (p = 0.038), using mobile for more than 6 h (p = 0.04), and using electronic devices while sitting on the floor (p = 0.02) were associated with a higher prevalence of neck pain among participants.

**Conclusion:**

In our study, we observed a relatively high prevalence of neck pain, which was linked to extended daily mobile phone usage and body posture during electronic device use. Policymakers may consider interventions aimed at reducing mobile phone usage and promoting proper body posture while using electronic devices as potential strategies to alleviate the burden of neck pain among Iranian adolescents.

## Background

Musculoskeletal pain (MSKP) is a global health concern that affects individuals of all ages and genders, presenting with diverse manifestations [1]. The prevalence of MSKPs has been on the rise in recent years. This increase can be attributed to the adoption of sedentary lifestyles, often involving prolonged periods of sitting [2, 3]. The World Health Organization (WHO) has ranked MSKP among the top ten contributors to disability-adjusted life years (DALYs) in adolescents aged 15–19 [4]. Neck pain, with a mean prevalence of 33.4%, is one of the most common MSKPs experienced by children and adolescents [5]. Although the exact underlying causes of neck pain in this age group remain unclear, certain factors, such as physical inactivity and psychological issues, have been identified as potential risk factors [6, 7]. Given the association between MSKP during childhood and adolescence, increased medication use, and a higher frequency of medical consultations, it is crucial to identify and effectively address potential risk factors contributing to neck pain and other MSKPs in this population [8].

Information and Communication Technology (ICT) comprises a wide range of devices, including mobile phones, tablets, gaming consoles, televisions, computers, and laptops. [9]. Recently, children and adolescents are devoting more time to using these ICT devices than ever, primarily due to their role in online education and accessibility [10]. It is estimated that children and adolescents spend an average of 5–7 h a day on their handheld devices, often with their heads inclined forward for reading and texting [11]. This extended and repetitive posture of forward head flexion, associated with gazing at screens of mobile phones and tablets, leads to a phenomenon known as “text neck syndrome,” resulting in cervical spinal degeneration [11]. The head’s weight on the spine increases during forward flexion, with the pressure on the neck depending on the extent and frequency of forward head bending [11]. Hence, excessive ICT use is suggested as one of the possible causes of neck pain in children and adolescents [11, 12].

Studies on the association between ICT use and MSKPs, especially neck pain among adolescents, are limited, and there are controversies regarding this association [2, 13, 14]. Moreover, most of these studies have been conducted in developed countries, and there is a lack of research in developing countries. Therefore, this study aimed to assess the relationship between exposure to common technology devices and reported neck pain symptoms among school-aged adolescents. We hypothesized that there is an association between technology device use and self-reported neck pain among adolescents.

## Method and materials

### Design

This cross-sectional study was conducted from June to October 2021. The ethics committee of Tehran University of Medical Sciences approved the study (ethics code: IR.TUMS.IKHC.REC.1400.320). The ethics committee waived the parental consent for this study as this was an observational study with no intervention.

### Participants & setting

We calculated the sample size using G*Power 3.1.9.7. Employing the Exact test family and a binomial statistical test, with a small effect size of 0.05, α = 0.05, and power = 0.8, we determined a sample size of 786 participants. To accommodate an anticipated dropout rate of 5%, we enrolled 808 individuals in our study [15, 16]. Inclusion criteria were: (1) age between 11 and 19, (2) being a student, (3) ability to read and write in Persian, and 3) willingness to participate in the study. Exclusion criteria were: (1) any history of cervical trauma, (2) history of diseases involving the neck region, such as rheumatic diseases and rheumatoid arthritis, and (3) history of severe systemic diseases.

In this study, we utilized a randomized multi-stage cluster sampling method. Initially, we randomly selected two city districts out of a total of three in Qazvin. Next, two schools were randomly chosen from each of these city districts, and a total of 202 students were randomly selected from each chosen school. To do this, we acquired the class lists and selected the first class from every three available. One of our researchers then attended the selected classes and gathered participants’ data through questionnaires. We explained the study’s objectives to the participants, and they provided informed consent before participating in the study.

### Measurements

Our questionnaire evaluated three groups of variables, including basic and demographic variables, exposures, and outcomes.

The demographic variables included age (years), gender (male or female), educational level (level one high school or level two high school), body mass index (BMI), and family structure (living with both parents, father, mother, or other relatives). Level one high school equals 7 to 9 years of education, and level two high school equals 10 to 12 years of education in Iran. The exposures (independent variable) included types of ICTs (e.g., mobile phone, tablet, PlayStation 4, laptop, and computer), position using electronic devices (sitting at the table, sitting on the floor, and lying), and the average amount of time they spent with each ICT per day in the past week (1. I do not use, 2. less than 3 h per day, 3. between 3 and 6 h per day, 4. more than 6 h per day). The outcome was any episodes of neck pain in the past week, as reported by the participants.

### Statistical analysis

Mean and standard deviation (SD) were calculated for continuous variables. Number and percentage were calculated for categorical variables. We used the Fisher exact or Chi-square tests to compare the categorical variables across groups. We used a binary logistic regression model to determine the factors independently associated with neck pain and calculated the adjusted odds ratios (AOR). We adjusted the model for BMI and gender as possible confounding factors. Missing values were excluded from the relevant analyses. P < 0.05 was considered statistically significant. We used IBM SPSS version 22 for all the analyses.

## Results

Eight hundred-eight adolescents aged 11 to 19 were included in our study. Our sample included 73.5% female participants, with a mean age of 15.1 ± 1.7 years, and 26.5% male participants, with an average age of 14.5 ± 1.5 years. Among all participants, 95.2% lived with both parents, while 3.3% lived with their mother, 0.6% with their father, and 0.9% with other relatives. The prevalence of neck pain was 21.5% in our study, with 174 participants reporting neck pain in the week before the study. The prevalence of neck pain was significantly higher among females than males (23.7% vs. 15.4%, p = 0.012), and it was also more common among students in level two high school compared to those in level one high school (25.2% vs. 17.4%, p = 0.01) (Table 1).

**Table 1:** Relationship between neck pain and demographic characteristics of participants

Variables		Neck pain			
		**Total**	**Yes**	**No**	**P-Value**
**Sex (n = 808)**	Male	214 (26.5)	33 (15.4)	181 (84.6)	0.012
	Female	594 (73.5)	141 (23.7)	453 (76.3)	
**BMI (kg/m2) (n = 759)**	Underweight (BMI < 18.5)	215 (28.3)	43 (20)	172 (80)	0.60
	Normal (18.5–24.9)	443 (58.4)	98 (22.2)	345 (77.8)	
	Overweight (25.0-29.9)	82 (10.8)	20 (24.4)	62 (75.63)	
	Obese (> 30)	19 (2.5)	6 (31.6)	13 (68.4)	
**Educational level (n = 744)**	Lower high school	442 (59.4)	77 (17.4)	365 (82.6)	0.01
	Upper high school	302 (40.6)	76 (25.2)	226 (74.8)	

Values are reported as numbers (percentages)

Mobile phones (96.9%), laptops (30.5%), and computers (23.4%) were the most frequently used devices among the participants. Duration of mobile phone use (p = 0.018), duration of PlayStation 4 use (p = 0.012), and position using electronic devices (p = 0.014) were associated with the prevalence of neck pain among the participants (Table 2).

**Table 2:** Association between neck pain and use of electronic devices among the participants

Variables		Neck pain No. (%)			
		Total	Yes	No	P-Value
**Mobile phone use (n = 768)**	No	24 (3.1)	1 (4.2)	23 (95.8)	0.018
	< 3 h	321 (41.8)	57 (17.8)	264 (82.2)	
	3–6 h	276 (36)	59 (21.4)	217 (78.6)	
	> 6 h	147 (19.1)	42 (28.6)	105 (71.4)	
**Tablet use (n = 744)**	No	663 (89.1)	140 (21.1)	523 (78.9)	0.69
	< 3 h	59 (7.9)	11 (18.6)	48 (81.4)	
	3–6 h	17 (2.3)	2 (11.8)	15 (88.2)	
	> 6 h	5 (0.7)	0 (0)	5 (100)	
**Computer use (n = 744)**	No	570 (76.6)	118 (20.7)	452 (79.3)	0.51
	< 3 h	146 (19.6)	27 (18.5)	119 (81.5)	
	3–6 h	21 (2.8)	6 (28.6)	15 (71.4)	
	> 6 h	7 (1)	2 (28.6)	5 (71.4)	
**Laptop use (n = 744)**	No	517 (69.5)	105 (23.3)	412 (79.7)	0.62
	< 3 h	180 (24.2)	37 (20.6)	143 (79.4)	
	3–6 h	33 (4.4)	7 (21.2)	26 (78.8)	
	> 6 h	14 (1.9)	4 (28.6)	10 (71.4)	
**Playstation 4 use (n = 744)**	No	631 (84.8)	134 (21.2)	497 (78.8)	0.012
	< 3 h	101 (13.6)	26 (25.7)	75 (74.3)	
	3–6 h	8 (1.1)	5 (62.5)	3 (37.5)	
	> 6 h	4 (0.5)	3 (75)	1 (25)	
**Position using electronic devices (n = 808)**	Sitting at the table	324 (40.1)	61 (18.8)	263 (81.2)	0.014
	Sitting on the floor	324 (40.1)	65 (20.1)	259 (79.9)	
	Lying	160 (19.8)	48 (30)	112 (70)	

Values are reported as numbers (percentage)

In the regression model, the female gender (AOR = 1.62, p = 0.038), using mobile for more than 6 h (AOR = 1.62, p = 0.04), and using electronic devices while sitting on the floor (AOR = 1.75, p = 0.02) were associated with a higher prevalence of neck pain among participants (Table 3).

**Table 3:** Results of binary logistic regression model on the association between exposures and confounders and neck pain among participants

Dependent variable	Independent Variables		Coefficient	Standard error	Adjusted odds ratio	P-value
**Neck Pain**	**Female gender**		0.484	0.234	1.62	0.038
	**BMI (kg/m2)**	< 18.5	Reference			
		18.5–24.9	0.09	0.21	1.09	0.67
		25–29.9	0.17	0.35	1.18	0.62
		>=30	0.35	0.62	1.42	0.57
	**Mobile use**	< 3 h	Reference			
		3–6 h	0.165	0.219	1.18	0.45
		> 6 h	0.472	0.251	1.63	0.04
	**Tablet use**	< 3 h	Reference			
		3–6 h	0.31	0.42	1.46	0.49
		> 6 h	0.13	0.57	1.14	0.89
	**Laptop use**	< 3 h	Reference			
		3–6 h	0.28	0.46	1.32	0.54
		> 6 h	0.02	0.76	1.02	0.97
	**Computer use**	< 3 h	Reference			
		3–6 h	0.804	0.543	2.23	0.13
		> 6 h	0.74	0.986	2.09	0.45
	**Playstation 4 use**	< 3 h	Reference			
		3–6 h	1.32	0.77	3.73	0.08
		> 6 h	0.69	1.15	1.99	0.54
	**Position using electronic devices**	Siting at the table	Reference			
		Sitting on the floor	0.56	0.24	1.75	0.02
		lying	0.09	0.22	1.1	0.6

## Discussion

MSKPs often lack definitive treatments and may require prolonged therapeutic interventions, resulting in significant adverse consequences affecting healthcare demands, daily functional abilities, and adolescent social engagement [1, 17]. Many of these disorders could potentially be prevented by identifying, mitigating, and managing risk factors [18]. Long-term use of technology devices, which is a possible risk factor for MSKPs, is very common among youth [19, 20]. The COVID-19 pandemic, characterized by extended periods of quarantine and widespread engagement in virtual classes among school-age adolescents, has led to a significant increase in the use of technology devices [21]. By investigating the association between neck pain and the use of technology devices, we can uncover a distinct pattern of adverse consequences related to excessive technology use [22, 23]. This study was one of the few studies in the context of a developing country on the factors associated with neck pain among school-age adolescents. In our study, the prevalence of neck pain was 21.5%. After adjusting for several variables, we found that factors such as female gender, using electronic devices while sitting on the floor, and using mobile phones for more than 6 h were associated with a higher prevalence of neck pain among Iranian school-age adolescents.

Among the devices we examined in our study, mobile phones emerged as the most commonly used. This finding is crucial as we discovered a significant association between using mobile phones for more than 6 h and a higher likelihood of experiencing neck pain among adolescents. In the study conducted by Mongkonkansai et al., the duration of smartphone use was a predictor for musculoskeletal discomfort among students. When comparing students who used cell phones for less than 60 min per day to those who exceeded this threshold, the latter group exhibited a tenfold higher likelihood of developing musculoskeletal disorders [22]. Considering that mobile phone use is common among adolescents and its excessive usage is associated with the development of neck pain, it becomes crucial to understand the reasons behind unnecessary mobile phone use among Iranian adolescents. Designing interventions aimed at reducing the time spent on mobile devices could be an effective preventive strategy to alleviate the burden of neck pain among Iranian adolescents.

We found that the body position while using electronic devices is associated with the likelihood of experiencing neck pain among adolescents. While the prevalence of neck pain was higher among those using devices while lying down, our regression model revealed that using electronic devices while sitting on the floor was associated with an increased likelihood of neck pain. This finding aligns with prior studies, which have indicated that prolonged use of electronic devices with improper posture throughout the day can elevate the risk of experiencing discomfort, pain, and fatigue [24, 25]. An uncomfortable sitting posture can also result in increased intradiscal pressure, potential disc malnutrition and pose risks to the musculoskeletal system [26]. Although we did not assess neck and head posture in our study, it’s worth noting that forward head posture is common among individuals addicted to smartphones, which could potentially lead to reduced endurance in extensor muscles [27]. This finding suggests that addressing and correcting uncomfortable neck postures during electronic device usage may be a viable strategy to alleviate or prevent neck pain among adolescents who frequently utilize such devices [28].

In our study, 21.5% of participants reported experiencing neck pain in the week leading up to the study. The prevalence of neck pain among children and adolescents varies considerably across studies, ranging from 12.3–32% [1, 29, 30]. Notably, the prevalence of neck pain tends to increase with age, and spinal pain during adolescence is linked to a higher risk of experiencing spinal pain in adulthood [5]. Consequently, the relatively high prevalence of neck pain observed in our sample may indicate potentially elevated neck pain rates among Iranians in the years to come. As a result, it is imperative for policymakers to focus on identifying and addressing the root causes and risk factors of neck pain among Iranian children from an early age to mitigate future consequences.

We found that female gender is associated with higher odds of neck pain in adolescents, which aligns with previous studies [1, 12, 31]. Several factors could account for this finding. It’s possible that boys generally have a higher pain threshold than girls, and hormonal changes related to early female puberty might play a role [32, 33]. Furthermore, the greater social acceptability for women to openly express their symptoms might also contribute to this finding [34]. Overall, the girls can be a group that may benefit from the interventions to mitigate the neck pain risk factors among Iranian adolescents and could be potential candidates for such interventions.

### Limitations

This study had a cross-sectional design, which is inappropriate for evaluating causal relationships between variables, and there is a need for future longitudinal studies to determine the risk factors of neck pain among Iranian adolescents. Furthermore, as this study was limited to Qazvin province, we may not generalize the results to the entire Iranian population, and there is a need for large-scale national studies in the future.

## Conclusion

The prevalence of neck pain was relatively high in our study and was associated with using mobile phones for more than six hours a day and the body position while using electronic devices. Considering that mobile phones are widely used electronic devices among adolescents, they could be a possible target for future interventions. These interventions could encompass educational efforts aimed at promoting proper posture while using electronic devices and strategies designed to reduce the amount of time spent on mobile phones, which can play a pivotal roles in alleviating the burden of neck pain among Iranian adolescents.

## Data Availability

The datasets generated and/or analysed during the current study are not publicly available to keep the confidentiality of data and privacy of participants, but are available from the corresponding author on reasonable request.

## Abbreviations

AOR: Adjusted odds ratio
DALYs: Disability-adjusted life years
ICT: Information and Communication Technology
MSKP: Musculoskeletal pain
